# Patients Receiving Palliative Care and Their Families' Experiences of Participating in a “Patient-Centered Family Meeting”: A Qualitative Substudy of the Valuing Opinions, Individual Communication, and Experience Feasibility Trial

**DOI:** 10.1089/pmr.2020.0109

**Published:** 2021-11-10

**Authors:** Philippa J. Cahill, Elizabeth A. Lobb, Christine R. Sanderson, Jane L. Phillips

**Affiliations:** ^1^ School of Medicine, University of Notre Dame Australia, Darlinghurst, New South Wales, Australia.; ^2^ Department of Palliative Care Research, Calvary Palliative and End of Life Care Research Institute, Calvary Health Care Kogarah, Kogarah, New South Wales, Australia.; ^3^ Faculty of Health, University of Technology Sydney, Ultimo, New South Wales, Australia.; ^4^ Palliative Care Facility, Territory Palliative Care, Alice Springs Hospital, Central Australia, Northern Territory, Australia.; ^5^ School of Nursing, Faculty of Health, Queensland University of Technology, Kelvin Grove, Queensland, Australia.

**Keywords:** family conference, family meeting, palliative care, patient centered, qualitative study

## Abstract

***Background:*** Family meetings are used in palliative care to facilitate discussion between palliative patients, their families, and the clinical team. However, few studies have undertaken qualitative assessment of the impact of family meetings on patients and their families.

***Objectives:*** To explore inpatients receiving palliative care and their families' experiences of participation in a patient-centered family meeting (“Meeting”), where the patient sets the Meeting agenda.

***Design:*** This qualitative study used the constant comparative method for thematic content analysis of the data.

***Setting/Participants:*** The setting was a specialist palliative care (SPC) inpatient unit in Australia. Nine palliative care inpatients and nine family members were interviewed.

***Measurements:*** Semistructured interviews were used evaluate the patients' and their families' experiences and perceptions of the Meeting.

***Results:*** Three overarching themes described the experiences of participating in a patient-focused family meeting, namely that the Meeting: (1) provides a forum for inpatients receiving SPC to speak openly about their end-of-life concerns, clarify issues, and is of comfort to patients; (2) provides the family members with a voice, and an opportunity to discuss their concerns and have their needs addressed; and (3) helps to ensure that everyone is “on the same page” and patient care plans can be discussed.

***Conclusions:*** These Meetings are a potentially effective means of supporting certain palliative care patients and their families to articulate, confront, and address end-of-life issues in the presence of the interdisciplinary team. It is important to undertake further research to further examine the evidence for this Meeting model and to identify the patients and families who would most benefit from this type of Meeting.

## Introduction

Optimal communication in palliative care is essential so that the concerns and needs of patients and families are identified and addressed.^1–3^ Meetings involving patients receiving palliative care, their family, and the interdisciplinary team (IDT) have been variously referred to as “family meetings” or “family conferences.”^4,5^ These terms are used interchangeably in both the clinical and research setting. Regardless of the terminology, these meetings in the specialist palliative care (SPC) setting aim to improve patient/family/team communication.^5–8^ They are also designed to facilitate discussion between patients receiving palliative care, their families, and the IDT about individual patient needs and concerns, care options and decisions, and end-of-life issues.^4,9^ In this context “family” may refer to whomever the patient nominates as “family,” or a family equivalent, such as a close friend and/or carer.^4^

In 2008, the *Guidelines for Conducting Family Meetings in Palliative Care* were developed in Australia.^5^ An evaluation of the effectiveness of these *Guidelines*^10^ demonstrated a significant increase in family members' unmet needs being satisfied as a result of participating in the meeting. The timing of the family meeting and a review of the benefits of patient participation were suggested as important areas for further research.^10^

While a recent study^11^ demonstrated that a family meeting resulted in unmet family needs and concerns being more effectively addressed, the authors recommended that the impact of family meetings be explored from the family and IDT perspectives.

This recommendation reflects the limited number of studies that have examined the impact of family meetings on patients and families in the palliative care setting.^10–15^ No studies have used validated quantitative measures to demonstrate benefits for patients attending family meetings, and the paucity of studies has undertaken qualitative assessment of the effect on patients attending a family meeting.^14,16^ A recent systematic review confirmed the lack of robust evidence to support the utility of family meetings in SPC.^17^

### Patient-centered family meetings

“Patient-centered” palliative care is critical as it focuses on the patient's quality of life^18^ and the imperative to address patient's end-of-life clinical, psychosocial, and spiritual concerns and needs in a timely manner. Patient-centered care may be defined as health care that is respectful and responsive to patients' preferences and needs while also acknowledging the individual patient's values.^19^ A key strategy for enhancing patient-centered care is to engage effectively with the patient's family and significant others.^19^ At its core, palliative care is about using an IDT approach to improve the quality of life of patients and their families facing end-of-life concerns.^20^ The principles of patient-centered care are therefore congruent with the clinical practice of palliative care.

A pilot project conducted in a specialist inpatient palliative care unit in New South Wales (NSW), Australia, provided qualitative findings that patient-centered family meetings enhanced the patient's active participation. Patients and families focused on end-of-life issues and articulated their concerns and demonstrated their care for each other.^14^

Family meetings involve considerable use of clinical resources and time. To enhance the body of knowledge related to family meetings in palliative care, the Valuing Opinions, Individual Communication and Experience (VOICE) Study was developed (ACTRN12616001083482). The VOICE Study was designed to assess the acceptability and feasibility of providing *planned* “Meetings” in specialist inpatient palliative care.^21^ “Patient-centered” care provided the conceptual framework for this Study. The proposed intervention—a planned patient-centered family meeting (“Meeting”) was designed to be patient centered. This aim was achieved at the time of the patient's admission by having the patients identify their key issues and concerns, which then formed the agenda for the Meeting. Textbox 1 summarizes the key Meeting components.

**Textbox 1.** Patient-Centered Family Meeting at the Intervention Site

1. The Meeting was offered to eligible patients during the first 10 days of an inpatient admission at a specialist palliative care service in metropolitan Sydney, New South Wales, Australia.
2. The patients identified the family member(s) they wished to attend the Meeting.
3. Before the Meeting, the patient formulated with the on-site research lead (P.J.C.) an agenda based on three key questions: (1) How do you see your health problems at the moment? (2) What do you expect from this admission? (3) Do you have any concerns about what is happening to you for which you would like help?
4. The patient-set agenda was provided to the interdisciplinary team participants before the Meeting.
5. The palliative care consultant and social worker routinely attended and facilitated the Meeting. Other clinicians participated when their specific expertise or advice was required based on the patient-set agenda.

The “patient-centered” component of the study, which is the novel aspect, focused on patients developing their own agenda for the Meeting. Previous evidence indicates that for many family meetings the agenda is set by the IDT.^11,22^ In this study, the questions that the patient often included in the agenda could include family issues and often did. The patient then nominated the family member(s) to attend. Hence, the primary focus was the way in which the patient prepared for the Meeting. However, this did not preclude family members from having an active role in either the preparation of the agenda or contributing their own questions and/or concerns during the Meeting. There were no limits imposed on the number of family members the patient invited to the Meeting.

This article reports on the experiences of patient and family Meeting participants, utilizing the COnsolidated criteria for REporting Qualitative research Guidelines.^23^ The data on the clinician's experience of these Meetings are reported elsewhere.^24^

## Aim

1. To explore inpatients' and their families' perceptions and experiences of participating in a patient-centered family meeting (“Meeting”) that used a patient-set agenda in an SPC unit.
2. To understand the benefits and burden of participating in a Meeting from the perspectives of patients and their families.

## Materials and Methods

### Design: qualitative substudy.

The VOICE Study protocol has been published elsewhere.^21^ This article reports on the qualitative substudy involving patients and their families.

### Setting

Before the study commencing, the researchers (P.J.C. and C.R.S.) provided clinicians who led the family meetings at the intervention site with education about the VOICE Study and a Meeting manual that outlined the key elements of the Meeting intervention. The research lead (P.J.C.) on-site had not previously worked with the clinicians at the participating site, while CRS had previously worked with one of the palliative care physicians.

### Recruitment/participants

Introductory research information was provided to all eligible inpatients and their families. To be eligible for inclusion in the VOICE Study the inpatient had to:

1. Be 18 years or older and admitted to the participating SPC inpatient facility within the last 7 days.
2. Have a terminal illness (a prognosis of ≤12 months but expected to live at least 14 days, which was the duration of the study protocol's intervention and follow-up).
3. Be able to identify a family member or a nominated equivalent person who consents to the study and Meeting so that the family component of the Meeting is fulfilled.
4. Be able to physically participate based on the Australian (Modified) Karnofsky Performance Scale (AKPS) with an AKPS score ≥30.^25^
5. Be able to read and speak English (as the study measures had been validated for English-speaking populations).
6. Be able to provide an informed consent.

Patients who were cognitively impaired due to disease or delirium were excluded.

All potential participants were informed that the VOICE Study formed part of the research lead's (P.J.C.) doctoral project and that the research was focused on understanding ways to improve how SPC can be provided to patients and families.

The family inclusion criteria were an adult who was 18 years or older and who:

1. Has a family member/friend who is a palliative care inpatient.
2. Is invited by the patient to participate in the Meeting.
3. Has English language and cognitive skills sufficient to complete baseline information and validated questionnaires and is able to contribute effectively in the Meeting.
4. Is able to provide informed consent.

The patient nominated which family member would be approached to participate in both the study and Meeting, however, other family members could participate in the Meeting.

### Research team

The female research team comprised the following: the Director of an Interdisciplinary Research Center (J.L.P.) who focuses on the aged and chronic disease and palliative care research; an experienced psychosocial palliative care researcher (E.A.L.); and a senior palliative care specialist and researcher (C.R.S.). The research lead (P.J.C.) was a PhD student who had studied qualitative and quantitative research methods at a tertiary level.

### Data collection process

The VOICE protocol stipulated that the patient interview occurred one to two days after the Meeting to optimize the patient's recall of the Meeting. The family interview occurred on day 14 of the patient's admission date.^21^ This protocol requirement was based on the average length of stay for NSW inpatient palliative care patients in 2014 being 13.4 days.^27^ This time frame ensured the likelihood of completing the family member's interview before patient discharge or death. The range of days for the patient interview was one to four days after Meeting and the range for family interviews was days 13–20 after patient admission. However, depending on the clinical condition of the patient and the availability of the family member, interview times were negotiated and sometimes deviated from the protocol.

All interviews were conducted on-site by the research lead (P.J.C.) between December 2017 and December 2018. Patients and family members who had participated in a Meeting were approached face-to-face for an individual interview. Interviews were conducted in a quiet space to ensure minimal disruption and accurate recording.

The semistructured interview sequence is detailed in Textbox 2. The questions were based on the pilot project interview sequence.^14^ They were designed to gain an understanding of the patients' and families' experience of the Meetings and identify the benefits and challenges of participating in these Meetings. This interview sequence for patients and their family members enabled the researcher at the intervention site to do the following: guide the line of questioning in a consistent manner and address the key issues of enquiry^28^; and to explore responses to gain a more nuanced understanding of the issues.

**Textbox 2.** Participant Interview Sequence

1. What was the family meeting like for you?
2. How did you feel after the family meeting (patient)?
3. Was anything that was talked about upsetting or distressing for you?
4. Were there other things you would like to have talked about at the meeting? (If the answer was “No,” go to question 6)
5. Why do you think you didn't talk about them?
6. Were you able to talk about how you were feeling at the family meeting?
7. Were you able to talk about your relationship and interactions with your family member at the family meeting?
8. Do you think the family meeting was helpful or not helpful for the family members who attended? Please tell me why…

Prompts were used to elicit further information, if suggested by the interviewee's response(s), being mindful of the interviewee's physical and emotional status. All consenting participants were only interviewed once. For all interviews, the interviewer (P.J.C.) concluded the interview when the participants had responded to all the questions and indicated they had no further contribution or comments to provide.

The majority of interviews (*n* = 14) were digitally recorded and transcribed verbatim. However, two patients and one family member for whom English was not their first language declined a recorded interview. They requested the interviewer (P.J.C.) record their answers manually. Another family member interview was also recorded manually. The researcher (P.J.C.) listened to the original recordings to check all transcripts, and completed field notes. The authors made the decision that transcripts were not returned for comment to the patients as this was challenging, given their symptom burden and life expectancy. Four patients died during their inpatient stay following completion of the study and two patients died 14 days postdischarge. For families, the benefit of checking the transcript was outweighed by the potential time burden for them with their other competing demands associated with the care of their relative.

### Data analysis

The data analysis was guided by the patient-centered interview schedule. Key elements of thematic analysis^25,29^ guided the development of themes using the coded datasets. The constant comparative method^23^ guided the development of themes derived from the data. Blinded coding of 22% of the transcripts was undertaken by three of the researchers (P.J.C., E.A.L., and C.R.S.) to classify initial codes. The researchers (P.J.C., E.A.L., and C.R.S.) discussed these codes and reached consensus. The research lead (P.J.C.) coded the other transcripts based on the initial codes. Data management did not include a coding tree or software. Data saturation was attained with conclusive categories identified for the coded data.^25^ Quotes to exemplify themes were deidentified.

### Ethics

St Vincent's Hospital Human Research and Ethics Committee approved the VOICE Study (Ref. No.15/SVH/33—SVH File No.: 15/021) on April 20, 2015.

## Results

**FIG. 1.**
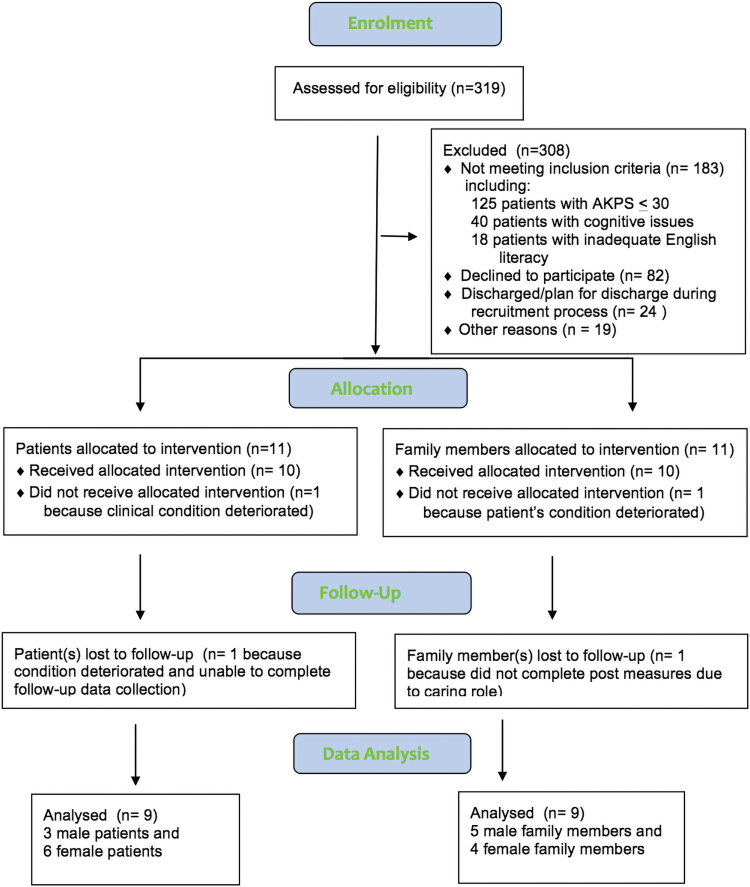
The screening and recruitment results at the intervention site. AKPS, Australian (Modified) Karnofsky Performance Scale.

Figure 1 summarizes the screening and recruitment results at the intervention site. Over a 10-month period, 319 patients were screened. Of the eligible patients, 26% (*n* = 82) declined to participate. The reason often provided was that the patients felt too unwell to do so, and 17 (21%) of these eligible patients died within 14 days.

In 40% of cases, family members contributed to the agenda before the Meeting. The mean patient age was 71 years (standard deviation [SD] = 7.16). The median family age group was 55–64 years. The mean recorded interview duration was 15.86 minutes (SD = 9.54). While the interviews were relatively brief, it should be noted that one interview question, if answered in the negative, required no further response. The brevity of several patient interviews contributed to the overall mean interview time. These patients did not provide detailed information and the interviewer did not press them for additional information as most had a high symptom burden such as fatigue. The research lead (P.J.C.) who undertook the interviews observed that while patients participated readily in the Meeting, several patients experienced the post-Meeting interview as burdensome and provided limited responses to the interview questions.

Three overarching themes described the experiences of participating in a patient-focused family meeting, namely that the Meeting: (1) provides a forum for inpatients receiving SPC to speak openly about their end-of-life concerns, clarify issues, and is of comfort to patients; (2) provides the family members with a voice, and an opportunity to discuss their concerns and have their needs addressed; and (3) helps to ensure that everyone is “on the same page” and patient care plans can be discussed.

### Patient cohort

*1. A patient-focused family meeting provides a forum for inpatients receiving SPC to speak openly about their end-of-life concerns, clarify issues, and is of comfort to patients.*

Patients identified that the Meeting model provided a forum where open and honest communication could occur between them and their significant family member(s) based on their prepared agenda:

… And I didn't want to open up the subject, in case, you know, it upset him [son] and you know we're both afraid (of) treading on one another's toes I s'pose, so … I thought it might have been rather harsh for him to hear it from me so because I was really facing facts about the whole illness and I thought well that might come across as a bit, you know, tough. (Patient 6)

This patient was able to raise end-of-life concerns at the Meeting with the son, and as a result:

… he had to process it in a fairly formal way and I think that was probably helpful for him and me … it started the ball rolling. And now that topic is open for, you know, discussion. (Patient 6)

The Meeting also consolidated how the patient wanted to generally approach end-of-life issues:

I thought it (the Meeting) was good. It's cemented … some of the things that we'd already talked about, … and it also cemented … the fact that I was happy to do things in blocks, instead of laying out the whole picture. I know there is a whole picture but I don't really want to know it all yet. (Patient 7)

In addition, the Meeting consolidated current issues:

… the family meeting was good because it consolidated a lot of things… (Patient 7)

Patients reported that the Meeting provided an opportunity to clarify what was happening to them at this time:

… you know, we all had a chance to ask and anything that we sort of didn't quite understand, it was clarified. I thought the meeting was excellent. (Patient 1)

The opportunity to talk with the clinicians and clarify their individual issues based on the patient-set agenda provided comfort to patients:

I always feel good after I talk to the doctors and you know clarify some of the things that you know I might have been thinking about over the last couple of days … I feel comforted after the meeting… I do feel like I've got the right team… (Patient 7)

As a result of speaking openly about their concerns, patients experienced an enhanced sense of peace:

I would say I'm more at peace than I was … I think just the whole process of having the questions asked and being heard and raising stuff. I honestly think it helps. (Patient 9)

One patient described the reassuring impact that the Meeting provided to raise specific end-of-life issues, which was a particular item on the agenda:

I tell you one thing that was really … mind-settling … knowing that (the patient's son) is not going to have to be racing around all over (name of city) trying to sort … funerals, that really sort of … detailed end-of-life sort of stuff. And that there is a morgue … I mean that sounds a bit weird but that's how I felt. (Patient 6)

### Family cohort

*2. A patient-focused family meeting provides the family members with a voice, and an opportunity to discuss their concerns and have their needs addressed.*

Family members indicated that the Meeting provided them with a voice to ask questions and express their concerns:

If I have question I want to ask I will raise it during the meeting … I feel free, so free to have my say… I think it's good to have our say and to participate in the family meeting so that the doctor can explain to us clearly about the outcome, what is their plan for the patient. (Wife of patient 2)

Family members also acknowledged that being able to contribute to the Meeting resulted in a balance between the patient and the family's contribution:

I mean I was almost thinking, “Alright good, that's enough air time for me guys” just focus on the patient now. Yeah it was well balanced I think. But yeah I certainly got a voice in that meeting which was good. (Son of patient 6)

The Meeting supported open discussion about end-of-life issues for the family:

It's like there's this really interesting interplay because you have a meeting with a doctor and they know, they can answer any question that you're brave enough to ask. I think a discussion around prognosis is important as much as you don't want to; I didn't find the prognosis shocking, … I found, it was confronting. It was just the fact that it was open and being talked about and being confirmed by someone who knows what they're talking about, rather than just my guesstimating. (Son of patient 9)

The patients had specifically included in their prepared agenda the need for a discussion about prognosis, so that the family had the opportunity to ask any questions and/or raise their concerns. For these families, the acknowledgment and discussion about the patient's prognosis were important. The impending wedding of one of the patient's adult children assisted the IDT members to forward plan the patient's care to facilitate the patient's attendance at the wedding:

… super useful … having … all the relative specialists … that was really useful and yes ‘X’ (has) been amazing, the social worker … just formulating a plan, yeah on how that'll work. (Son of patient 9)

Patients acknowledged that the Meeting met the families' need to express their concerns:

It was good for him [husband] to talk about all the things that he's been thinking about… all those pressures and stresses and strains that he's been under … It just got it ‘out in the open’ for him. (Patient 5)

Patients were comforted and grateful that family members were able to raise their issues and receive worthwhile responses from the IDT members present:

… the family meeting was just brilliant because for [daughter's name], she couldn't get answers but also for me, the anxiety about, you know, what if I get home and I can't cope? And each time with something like that, it just puts more stress on ‘X’ [daughter], so both [daughter], and myself, we thought the meeting was just fabulous and ‘X’ [social worker] was just wonderful. (Patient 1)

The IDT members' attendance at the Meeting also provided the family with a clear understanding of the different disciplines and their role in the care of the patient. In the palliative care setting, this was reassuring and informative for the family member:

And (it) was the opportunity to see all of the professionals in one room at the time. And I think we came away with a clear understanding of what everybody does, who has the authority over particular areas and that seems clear-cut, and how decisions are made. (Husband of patient 10)

The presence of the IDT members listening to the patient and indicating that they would continue to care for the patient during the end-of-life phase was also comforting for the family member who attended and witnessed this discussion:

They were the same questions coming up time and time again… except this time, ‘X’ [Palliative Care Physician] just sat there and just said, “Well this is what it is, this is where we can help, you know where we can guide you or whatever.” So yes it was … probably a more controlled environment too, because people weren't rushing, to get to the next person, … they had time just to sit and listen to her … it was positive that it was all about what she was about … And all about addressing her fears and problems…. You know that they're not going to abandon her, and just pass her on to somebody else. (Cousin of patient 7)

*3. A patient-focused family meeting helps to ensure that everyone is “on the same page” and patient care plans can be discussed.*

Patients reported that the Meeting contributed to the families' understanding of their current situation:

… she [patient's cousin] found it was helpful because it just showed that we were all on—we were all able to be “on the same page” when it came to, you know, what I wanted. (Patient 7)

The Meeting also provided the forum for families to discuss difficult issues so they could begin to move toward being “on the same page”:

Well I think he would not ask questions that I would—you know, because frightened, he would be a bit afraid that he'd be treading on my toes, especially the really detailed end-of-life stuff … (Patient 6)

Interviewer: Yes, so the Meeting allowed you to ask those questions?

Yes and in front of him was I think very valuable because we're both now getting towards the same page on that, on those details … (Patient 6)

Several patients included as an agenda item the need for a management plan or update on symptom issues. As a consequence, family members recounted that an outcome of the Meeting was a clearly articulated care plan:

I mean, it was in the middle of a meeting we actually got a structure that says, ‘Your next goal is to get up to the top floor”(Rehabilitation Ward), and then you've got … a structured three-week program. And then you do a big assessment and if you haven't improved, then you've got to worry about where you're going to go from here, and that's where, I needed to know that. … because when you walk in the door here … no one actually sits down and says, now, “This is why you're here and this is where we're going.” And so if the meeting was the only vehicle of getting that out and that was the purpose of it, that's fantastic. (Husband of patient 5)

The families' need for information about the future plan and consolidating this plan was often raised as an important outcome of the Meeting:

…it was helpful for my wife—my wife knows the future direction—I can go to a “good” nursing home—she wants me to be comfortable.

… it was helpful for my daughter—my daughter thought (the Meeting) was very good; she listened to everything (via mobile phone) and had more information. (Patient 2)

## Discussion

This qualitative study provides insights into the value and experiences of SPC inpatients and their families participating in a patient-centered approach to family meetings. The screening-to-recruitment ratio highlights the difficulties of recruitment to a research study in a palliative care setting. Patients may be too sick to participate, provide informed consent, or live long enough for study completion.^30^ Patients may also deteriorate or die before the Meeting can be scheduled. However, for patients who are well enough and desire to communicate their thoughts, feelings, and concerns in a family meeting, this type of Meeting is highly valued. For these patients, a Meeting makes it easier to discuss end-of-life issues that they have found difficult to raise with family outside a structured forum. This finding is also reflected in the pilot study results, where end-of-life issues were raised in over two-thirds (69%) of patient-centered meetings.^14^

To improve the participation rate, and to give patients and their families an opportunity to attend a Meeting, consideration could be given to these Meetings being offered to patients and families earlier in the disease trajectory. This may require a Meeting before admission to an SPC unit. Such a strategy may well address the issue of patients who, because of their symptom burden or short life expectancy, are unable/unwilling to participate. An alternative to this approach, for patients who are not physically able to participate, is to consider a Meeting with the family caregivers and the IDT. This provides families with a “voice” to discuss their concerns and have their needs addressed in a similar way to that described in this study's findings.

Similar to the pilot study,^14^ patients in this study also reported that they “felt better” in various ways as a result of participating in the Meeting. The patient who referred to the “mind-settling” impact of discussing specific end-of-life issues about the management of the body after death, and who also felt an enhanced sense of peace post-Meeting, exemplified this finding. The deliberate and conscious decision to enable the patient to set the agenda is designed to give the patient “agency” before the Meeting. It may be that this sense of agency enabled the patients to identify, and then speak frankly and openly about their end-of-life concerns, knowing that they had prepared their questions and had nominated which family member(s) they wished to participate in the Meeting.

Family members also identified that the Meeting provided them with a “voice” and a forum for their needs to be expressed and addressed. Having a “voice” is a critical element in contributing to the quality of the families' experience of their family member's death in a hospital setting because it can impact on several factors families considered important to the quality of this experience. These factors include the following: (1) the family's relationship with the health care provider; (2) identification of the person whom the family members can obtain answers for their concerns; and (3) what to expect about the course of the patient's disease.^31^ Families interviewed in the current study provided similar responses to those previously reported,^31^ such as knowing who to go to with a problem, having a clear care plan for their family member, and receiving an explanation of potential outcomes for their family member.

The findings from the family members in the current study also replicate the findings of another Australian study evaluating the impact of family meetings on family members in an inpatient palliative care unit.^13^ Families in this study reported that as result of the family meeting they felt heard, they obtained a better knowledge and relationship with the IDT members, and they were able to ask about prognosis and discuss this at the family meeting.^13^ Families in the current study report similar experiences. For the majority of family members, there was a strong sense that they too were able to articulate their concerns and issues, and participate actively in the Meeting discussion. They also gained a greater understanding of the role of the IDT members and were given meaningful responses by the IDT at the Meeting.

The experience of everyone being “on the same page” is illustrative of the results from the pilot study.^14^ The strength of the family meeting as a forum for communication is based upon the bringing together of the patient, the family (as nominated by the patient), and the IDT members. This enables discussion of the patient's issues and concerns, and participants hear information contemporaneously.^4^ The end result is that attendees have a sense of being “on the same page.” The participants simultaneously witness during the Meeting the verbal and nonverbal exchanges among the attendees, which are so important to all at this vulnerable time.^32^ The observation of nonverbal cues enhances the participants' understanding of the concerns and issues being expressed.^32^ The qualitative results of a previous study examining palliative care family conferences also concluded that the concurrent participation of attendees at a family conference led to participants being “on the same page.”^6^

In this study, patients and families required English fluency and literacy. However, in a clinical environment with no research imperative, interpreters can be used to enable these patient and family cohorts to participate in a Meeting.

As with all qualitative studies, there are limitations. We acknowledge that while the sample size is small, it is an acceptable size given the qualitative nature of the study design and the fact that data saturation was reached. This small sample size also reflects the acuity and the fluctuating clinical status of many palliative care patients. As only a small proportion of eligible patients consented to participate, the Meeting format may only be of interest to a subsection of the palliative care patient population. However, this requires further verification. As this is a qualitative study, the findings cannot be generalized.

### Strengths and limitations

To our knowledge, this is the first qualitative research study undertaken in an inpatient SPC context to evaluate patients' and families' experiences of family meetings, using the same semistructured interview sequence. Previous studies have reported data derived from interviews with family members, or in one study also interviewed patients about their Meeting experience.^14^ However, in this study, the patient interview questions differed to the feedback sought from the family members.^14^ The unique aspect of this study is the use of the same semistructured interview questions for patients and families. This enabled exploration and comparison of the Meeting experiences of both these participant groups.

However, there are limitations in this study. We have already mentioned the small sample size. In addition, the mean interview time was relatively brief largely because of the frailty of some patients who did not provide expansive answers to the questions. Given that these patients and families were a vulnerable cohort, the interviewer did not unduly probe patients or families when their answers were brief.

### Implications for research and practice

The research lead (P.J.C.) interviewed a small subsample of patients receiving SPC who desired to meet with the IDT and their families, but all patients were physically compromised and unable to give lengthy interviews. Hence, caution is required in interpreting the data. Based on the feedback from these patients and families, these Meetings are a potentially effective means of supporting certain palliative care patients and their families to articulate, confront, and address end-of-life issues in the presence of the IDT. The strength of these Meetings is in creating and promoting a shared conversation and understanding of the patients' disease, their prognosis, and end-of-life concerns. This may also lead to ongoing productive discussion between the patients, families, and the IDT as the end of life approaches.

Additional research should be undertaken to further examine the evidence for this Meeting model, and its generalizability and applicability in the palliative care context. The use of a validated quality-of-life measure pre- and postmeeting to evaluate the impact of these Meetings would be an important consideration in future research. Research of this nature could also delineate those patients and families for whom such Meetings would provide both short-term positive outcomes and also longer term family benefits postbereavement. Furthermore, the potential risks and burdens of meetings should be considered.

## Conclusions

In this study, the patients and families interviewed about the Meeting model reported beneficial and supportive experiences. The patient-set agenda enabled the patients to identify and discuss psychosocial, emotional, and relationship issues and concerns related to their current condition and end-of-life preparation. For some patients, these Meetings were critical for beginning the difficult conversations with their family that they wished to have before their death, often to resolve outstanding concerns. To enhance the uptake and provide patients and families with an opportunity to participate in this Meeting model, consideration can be given to offering this Meeting earlier in the disease trajectory. When patients are physically compromised, an alternative is for the family, as proxy decision makers for the patient, to meet with the IDT to ensure that both patient's and family's needs and concerns are raised and discussed.

We have previously noted that this Meeting model is not feasible for all patients.^24^ This observation was based on the recognition that there were finite clinical resources and time required to provide them as standard care, and that not all patients would require or be suitable for this Meeting model. It is important to establish with greater clarity any causal link between the Meeting and the patients' and families' experience. Further research is also required to identify which patients and families would receive either a benefit from this type of Meeting or (potentially) consider this type of Meeting a burden.

## Data Management and Sharing

Raw data are included in the article in the form of excerpts from the recorded interviews. All data were deidentified. The patient and family names, identification numbers, digital audio recordings, and transcripts were saved on a password-protected computer at the subacute facility. The corresponding author may be contacted for further information.

## Abbreviations Used

AKPS: Australian (Modified) Karnofsky Performance Scale
IDT: interdisciplinary team
NSW: New South Wales
SD: standard deviation
SPC: specialist palliative care
VOICE: Valuing Opinions, Individual Communication and Experience
